# Does clinical equipoise apply to cluster randomized trials in health research?

**DOI:** 10.1186/1745-6215-12-118

**Published:** 2011-05-11

**Authors:** Ariella Binik, Charles Weijer, Andrew D McRae, Jeremy M Grimshaw, Robert Boruch, Jamie C Brehaut, Allan Donner, Martin P Eccles, Raphael Saginur, Monica Taljaard, Merrick Zwarenstein

**Affiliations:** 1 Rotman Institute of Philosophy, Department of Philosophy, University of Western Ontario, London, ON, N6A 5B8, Canada; 2 Department of Medicine, University of Western Ontario, 339 Windermere Road, London, ON, N6A 5A5, Canada; 3 Department of Epidemiology and Biostatistics, University of Western Ontario, London, ON, N6A 5C1, Canada; 4 Division of Emergency Medicine, University of Calgary, Foothills Medical Centre, 1403 29th Street NW, Calgary, AB, T2N 2T9, Canada; 5 Ottawa Hospital Research Institute, Clinical Epidemiology Program, Civic Campus, 1053 Carling Avenue, Ottawa, ON, K1Y 4E9, Canada; 6 Department of Medicine, Faculty of Medicine, University of Ottawa, Ottawa, ON, K1H 8L6, Canada; 7 Graduate School of Education and Statistics Department, Wharton School, University of Pennsylvania, 3700 Walnut Street, Philadelphia, PA, 19104, USA; 8 Department of Epidemiology and Community Medicine, University of Ottawa, Ottawa, ON, K1H 8M5, Canada; 9 Robarts Clinical Trials, Robarts Research Institute, London, ON, N6A 5K8, Canada; 10 Institute of Health and Society, Newcastle University, 21 Claremont Place, Newcastle upon Tyne, NE2 4AA, UK; 11 Department of Medicine, University of Ottawa and Ottawa Hospital, Ottawa Hospital Research Institute, 1053 Carling Avenue, Ottawa, ON, K1Y 4E9, Canada; 12 Centre for Health Services Sciences, Sunnybrook Health Sciences Centre, 2075 Bayview Avenue, Toronto, ON, M4N 3M5, Canada

## Abstract

This article is part of a series of papers examining ethical issues in cluster randomized trials (CRTs) in health research. In the introductory paper in this series, Weijer and colleagues set out six areas of inquiry that must be addressed if the cluster trial is to be set on a firm ethical foundation. This paper addresses the third of the questions posed, namely, does clinical equipoise apply to CRTs in health research? The ethical principle of beneficence is the moral obligation not to harm needlessly and, when possible, to promote the welfare of research subjects. Two related ethical problems have been discussed in the CRT literature. First, are control groups that receive only usual care unduly disadvantaged? Second, when accumulating data suggests the superiority of one intervention in a trial, is there an ethical obligation to act?

In individually randomized trials involving patients, similar questions are addressed by the concept of clinical equipoise, that is, the ethical requirement that, at the start of a trial, there be a state of honest, professional disagreement in the community of expert practitioners as to the preferred treatment. Since CRTs may not involve physician-researchers and patient-subjects, the applicability of clinical equipoise to CRTs is uncertain. Here we argue that clinical equipoise may be usefully grounded in a trust relationship between the state and research subjects, and, as a result, clinical equipoise is applicable to CRTs. Clinical equipoise is used to argue that control groups receiving only usual care are not disadvantaged so long as the evidence supporting the experimental and control interventions is such that experts would disagree as to which is preferred. Further, while data accumulating during the course of a CRT may favor one intervention over another, clinical equipoise supports continuing the trial until the results are likely to be broadly convincing, often coinciding with the planned completion of the trial. Finally, clinical equipoise provides research ethics committees with formal and procedural guidelines that form an important part of the assessment of the benefits and harms of CRTs in health research.

## Introduction

This article is part of a series of papers examining ethical issues in cluster randomized trials (CRTs) in health research. CRTs are used increasingly in knowledge translation research, quality improvement research, community based intervention studies, public health research, and research in developing countries. While a small and growing literature explores ethical aspects of CRTs, cluster trials raise difficult issues that have not been addressed adequately. In the introductory paper in this series, Weijer and colleagues set out six areas of inquiry that must be addressed if the cluster trial is to be set on a firm ethical foundation [1]. These include identifying research subjects, obtaining informed consent, the applicability of clinical equipoise, benefit-harm analysis, the role and authority of gatekeepers, and the protection of vulnerable populations in CRTs. This paper addresses the third of the questions posed, namely, does clinical equipoise apply to CRTs in health research?

In the introductory paper of the series, Weijer and colleagues set out a standard framework of research ethics with four ethical principles: respect for persons, beneficence, justice, and respect for communities [1]. Beneficence is the moral obligation not to harm needlessly, and when possible, to promote the welfare of research subjects. In the context of clinical research, beneficence gives rise to the moral obligation to provide research subjects with a reasonable balance of harms and benefits. Establishing what constitutes a reasonable balance of harms and benefits is one of the central challenges in research ethics oversight [2].

Evaluating the balance of harms and benefits raises unique concerns for CRTs, which randomize groups or intact social units rather than individual subjects. In individually randomized controlled trials (RCTs), an acceptable balance of harms and benefits relies, in part, on the existence of clinical equipoise. Clinical equipoise refers to a state of honest, professional disagreement among the community of experts about the preferred treatment [3]. While clinical equipoise is central to the evaluation of harms and benefits in RCTs, its application to CRTs is uncertain and requires specification. In the following, we argue that clinical equipoise can be applied to the resolution of ethical tensions in the assessment of harms and benefits in CRTs.

We begin by identifying the central challenge in applying clinical equipoise to CRTs. Clinical equipoise is commonly understood as an ethical requirement emerging from the trust relationship between physician-researchers and patient-subjects. As such, the moral requirement for clinical equipoise presupposes the existence of a relationship between a physician-researcher and a patient-subject. Unlike RCTs, CRTs often do not involve relationships between physician-researchers and patient-subjects. In CRTs, the units of randomization may be schools, communities, or physician practices. As a result, clinical equipoise is not obviously applicable to CRTs.

To resolve this problem, we identify a trust relationship between the state and research subjects, in addition to the widely recognized trust relationship between a physician-researcher and the patient-subject. Recognizing clinical equipoise as emerging from the relationship between the state and research subjects situates clinical equipoise in a framework which can be applied to harm-benefit assessments in CRTs.

Our analysis proceeds in the following six steps. First, we identify difficulties associated with the evaluation of harms and benefits in CRTs and explore their similarity to ethical problems arising in RCTs. Second, we consider the role of clinical equipoise in resolving tensions associated with harm-benefit assessments in RCTs. Third, we elucidate the difficulties associated with applying clinical equipoise to CRTs. Fourth, we critically analyze the solutions proposed for resolving ethical tensions in CRTs. Fifth, we argue that recognizing a trust relationship between the state and research subjects provides a framework in which clinical equipoise can be applied to CRTs. In the sixth and final section, we explore the implications of implementing clinical equipoise as a moral requirement for CRTs with reference to an example CRT.

## Harm-benefit assessments in CRTs and RCTs

### Harm-benefit assessments in CRTs

A widely recognized ethical principle requires that all research offer participants a reasonable balance of harms and benefits [4]. This moral requirement aims to ensure that subjects will not be disadvantaged by their random intervention assignment. While most agree that subjects should not be disadvantaged by their random intervention assignment, implementing this requirement raises difficult ethical questions. For example, how should harms and benefits be calculated? What constitutes an acceptable balance of harms and benefits? What benefits, if any, are owed to control group subjects? Are there criteria for reviewing the acceptability of harm-benefit assessments?

Currently, the intricacies of harm-benefit assessments receive little attention in the literature on CRTs [5]. Most commentators overlook these difficulties and those who raise problems with harm-benefit assessments do not provide principled resolutions. Furthermore, commentators have not adequately addressed whether difficulties in harm-benefit assessments are unique to CRTs or akin to those encountered in RCTs. In the following section, we identify the concerns associated with harm-benefit assessments raised in the CRT literature and consider whether these are unique to CRTs or represent broader ethical concerns for clinical research involving human subjects.

Two ethical problems associated with harm-benefit assessments have been discussed in the CRT literature. The first ethical concern addresses the morally appropriate treatment of subjects in the control group of CRTs. Commentators argue that exposing subjects in a control group to an intervention hypothesized to be inferior or to no intervention at all may violate the ethical requirements of beneficence [6,8]. Klar and Donner argue that the considerable effort involved in participating in a CRT should not be left uncompensated [8]. More specifically, the ethical requirement to provide subjects with a reasonable balance of harms and benefits may not be fulfilled in CRTs in which control group subjects typically "receive only usual care" [8]. Similarly, Glanz and colleagues argue that control group subjects "may be burdened disproportionately by data collection requirements without receiving the benefit of services or resources" [6]. In other words, these authors raise concerns that the risks of participating in the control group of a CRT, which receives either no intervention or an intervention hypothesized to be inferior, may not stand in reasonable relation to the potential benefits of participation [6].

The second ethical concern arises from the preliminary results of a CRT, revealed during interim analysis [6,7,9]. While a trial is underway, the accumulation of data often suggests the superiority of one intervention over another. Commentators on the ethics of CRTs are concerned about the obligations of researchers when faced with these interim results. Glanz and colleagues argue that there is an ethical obligation to act on interim findings. Interim analyses can bring to light unforeseen and serious adverse effects or indicate that one arm of the trial is superior. In the former case, it would be unethical to continue the trial without modification to the intervention and in the latter case, the preferable treatment should be offered to all groups or individuals participating in the trial [6]. Another commentator claims that the decision to terminate a trial early based on interim findings is particularly complicated in CRTs because the statistical inefficiency of the cluster design complicates judgments about when sufficient evidence exists to ascertain trial benefits or harms [7].

### Similar concerns in RCTs

The commentators raise important concerns associated with harm-benefit assessments. A brief examination of the early literature on the ethics of individually randomized controlled trials reveals that the ethical concerns described above are not unique to CRTs. Parallel ethical concerns have been addressed with respect to RCTs.

For example, Ware (1989) raises concerns about the ethical obligations of physician-researchers to subjects when accumulating evidence suggests the superiority of the experimental intervention. He argues that even though evidence for the efficacy of extracorporeal membrane oxygenation (ECMO), an experimental intervention for the treatment of persistent pulmonary hypertension in newborns, would have been stronger in a trial involving concurrent control groups, ethical concerns led him to select an adaptive randomization design [10]. Ware's concern that "continuing to randomize infants when accumulating evidence strongly suggested the superiority of one treatment" unduly deprives subjects of potential beneficial experimental interventions [10] is mirrored in the commentary on ethical problems in CRTs.

Similarly, Shaw and Chalmers (1970) raise ethical concerns about the data accumulation problem akin to those raised in the CRT literature. They argue that physician-researchers can offer patients trial enrollment only when they are uncertain about the merits of one intervention over another. However, this uncertainty is often disturbed before a trial can be undertaken [11]. Clinical trials are developed to investigate the success of treatments which show promise in pilot trials; however, successful preliminary results worth testing in clinical trials seem to disturb an investigator's uncertainty before potential subjects could be approached for enrollment. In other words, the accumulated data from pilot trials provide physician-researchers with good reason to believe in the success of the new intervention [11]. As a result, a physician-researcher is ethically required to provide patients with the superior treatment.

Furthermore, the data accumulating during a trial raise ethical concerns. During the course of a trial, the accumulating data often show a tendency for one intervention to be more successful than another. These data may provide a physician-researcher with reason to believe in the superiority of one treatment. However, knowing about the superior treatment makes it impossible for the trial to proceed ethically [11]. As a result, ethical concerns seem to suggest that randomization must stop before a trial has run to completion [11]. In short, the ethical difficulties raised by harm-benefit assessments in CRTs are reflected in the early literature on the ethics of RCTs.

## Moral solution in RCTs: clinical equipoise

Given the similarity between the ethical problems raised by harm-benefit assessments in RCTs and CRTs, we look to the literature addressing the ethics of RCTs to inform our analysis of harm-benefit assessments in CRTs. The ethical problems raised in the early literature on RCTs are addressed by Freedman's notion of clinical equipoise. Clinical equipoise exists when there is a state of honest, professional disagreement in the community of expert practitioners as to the preferred treatment [3]. In other words, the ethical permissibility of comparing interventions in RCTs depends on disagreement among expert practitioners about the relative merits of the treatment alternatives. The existence of a state of clinical equipoise is a necessary ethical condition for the commencement of a trial.

Clinical equipoise is a necessary condition for the ethical justification of an RCT; however, it is not a sufficient condition. Clinical equipoise describes the conditions under which a research ethics committee can approve a trial but it does not fully explain a physician-researcher's duty of care to patients who have been identified, approached, asked for participation or enrolled in a trial. A physician-researcher's duty of care to patients also relies on a clinical judgment principle, which provides additional protections for patients involved in research [12]. According to the clinical judgment principle, a physician-researcher may not offer a patient enrollment or continued participation in a trial when the physician judges that it would be medically irresponsible to do so, and the evidence for this judgment is likely to be convincing to colleagues [12].

The clinical judgment principle requires physician-researchers to continue to exercise their professional judgment about particular patients. Consequently, it mitigates the concern that clinical equipoise seems to permit a physician-researcher to randomize a patient even when this may conflict with his or her professional judgment. Thus, the ethical justification of a trial relies on both the existence of a state of clinical equipoise and physician decision making in accordance with the clinical judgment principle. Clinical equipoise provides the moral guidance for an ethics committee and the clinical judgment principle guides physician-researchers in their decisions regarding the enrollment and continuation of particular patients in research.

Clinical equipoise is one of the most fundamental and widely cited concepts in research ethics. The central importance of clinical equipoise can be seen in its prominent appearance in the research ethics literature and in policies and guidelines governing the protection of human subjects in research [12]. For example, the U.S. National Bioethics Advisory Commission's recommendations for reform of federal regulations prescribing standards for institutional review boards (IRBs) recommends the use of clinical equipoise as a central concept in harm-benefit assessments [12,13]. In addition, clinical equipoise is centrally involved in controversial debates. For example, both critics and proponents of the use of placebos in HIV research in developing countries cite clinical equipoise as a moral requirement [14,15]. The requirement for clinical equipoise has become widely accepted as the moral justification for random treatment assignment in RCTs [12].

Clinical equipoise is widely endorsed and applied in the justification of RCTs; however, it has also been the center of controversy in the research ethics literature. Commentators disagree as to whether the concept of clinical equipoise is useful for the ethical justification of a trial, and they differ as to whose uncertainty ought to define the state of equipoise. Franklin Miller and Howard Brody reject the utility of clinical equipoise. They argue that clinical research and clinical practice have fundamentally different goals and that the rules governing each should not overlap [16]. On this understanding of clinical research, there is no fiduciary relationship between a physician-researcher and a patient-subject. Miller and Brody conclude that clinical equipoise--which is rooted in the duty of care a physician-researcher owes to a patient-subject--confuses the ethics of clinical practice with clinical research; it is unnecessary and should be abandoned [16].

Paul Miller and Charles Weijer have responded to Miller and Brody's argument by pointing out that many norms--such as the prohibition of murder and fraud--apply across diverse activities [17]. Further, denying that physicians have obligations to patients during research requires a difficult and undesirable kind of moral dissociation for physicians; they must ignore their professional obligations to their patients during research [17]. Moreover, Miller and Weijer have articulated a moral and legal foundation for the existence of a fiduciary relationship--and corresponding duty of care--between physician-researchers and patient-subjects [17,18].

Another group of scholars endorses clinical equipoise but offers diverse interpretations about its meaning. Within this group, some commentators interpret equipoise as an evidentiary standard while others interpret equipoise as uncertainty. The latter group, as elucidated by Djulbegovic, understands equipoise as existing at multiple levels [19]. For example, equipoise may exist at the level of subjects [20], physicians [21] or the community [22,23]. These different levels of equipoise may motivate different questions concerning the ethical justification of research [19]. However, in the current paper, clinical equipoise is understood as an evidentiary standard that provides guidance to research ethics committees. A critical exchange concerning the merits of different interpretations of clinical equipoise can be found elsewhere in the research ethics literature [24].

Clinical equipoise helps to resolve the ethical tensions raised by harm-benefit assessments in RCTs by providing a principled justification for the ethical permissibility of a trial. More specifically, it provides research ethics committees and researchers with procedural and substantive guidelines for their decision-making processes. Procedurally, a research ethics committee determines whether clinical equipoise obtains by analyzing the study protocol and justification, reviewing relevant literature, and, if necessary, by consulting with independent clinical experts [2]. Moreover, meeting the moral requirement of clinical equipoise requires that research ethics committees determine whether there is sufficient warrant to expose people to an experimental intervention and whether the proposed trial exposes subjects in the control arm to the risk of substandard intervention. The moral requirement for clinical equipoise is met if the research ethics committee finds that the evidence supporting the various treatment options is sufficient that, were it widely known, expert practitioners would disagree about the preferred treatment [2].

Clinical equipoise helps to resolve many of the ethical problems raised by RCTs. For example, it provides a procedure for establishing whether Ware's concern that a trial with a control group would unduly deprive control group subjects of the benefits of an experimental treatment is justified. More specifically, if a research ethics committee establishes that other expert practitioners have good grounds to believe that standard treatment is preferable to the experimental treatment, then control subjects would not be unduly disadvantaged in being deprived of the experimental intervention. In other words, control and intervention groups would both receive competent medical care.

Similarly, clinical equipoise provides a solution to Shaw and Chalmers' data accumulation problem [11]. While data accumulating prior to and during the course of a trial may seem to favor one treatment over another, clinical equipoise persists until the results are powerful enough to influence the judgment of the community of experts. Once the accumulating data are sufficient to disturb equipoise, the trial should be stopped. This type of evidence is most often generated at the end of a trial. It is widely recognized that decisions for early termination of a trial based on interim results are complicated [25]. Trends favoring one intervention over another often emerge early in a trial only to disappear later in the trial. As a result, overreacting to emerging or non-emerging trends in interim outcomes can result in incorrect conclusions [25].

Consequently, when accumulating evidence suggests the superiority of one intervention, it may be ethically permissible to continue a trial to completion given that the evidence necessary to disturb equipoise is most often generated only at the end of a trial. Finally, meeting the requirement of clinical equipoise ensures that all arms of a trial are consistent with competent medical care, defined as practices accepted by the community of medical experts [17].

## Does clinical equipoise apply to CRTs?

Freedman's clinical equipoise helps to resolve the ethical tensions associated with harm-benefit assessments and provides a principled ethical foundation for the permissibility of RCTs. As a result, a concept like clinical equipoise could help to resolve the similar ethical concerns in CRTs. However, it is not clear that clinical equipoise can be applied to CRTs. In the following section, we explore the challenges in applying clinical equipoise to CRTs.

The central challenge in applying clinical equipoise to CRTs stems from the origins of clinical equipoise in the duty of care a physician-researcher owes to all patient-subjects. Clinical equipoise is commonly understood as emerging from the relationship between a physician-researcher and a patient-subject [17]. On this understanding, clinical equipoise presupposes and requires the existence of relationships between physician-researchers and patient-subjects. Unlike RCTs, CRTs often involve no such relationships and as such, clinical equipoise is not obviously applicable as a moral requirement for CRTs.

A brief discussion of the nature of the relationship between physician-researchers and patient-subjects will help to elucidate why clinical equipoise is commonly thought to emerge only from the relationship between a physician-researcher and a patient-subject.

The relationship between physician-researchers and patient-subjects is best understood as a fiduciary relationship [18,26,27]. Fiduciary relationships are a distinctive type of trust relationship characterized by inequality [18]. In these relationships, one party (the beneficiary) lacks the abilities necessary to effectively protect and promote his or her own interests and consequently entrusts a more powerful party (the fiduciary) with discretionary power over the beneficiary's practical interests [12]. As a result of this entrustment, the beneficiary becomes dependent on the judgment and care of the fiduciary. The beneficiary's authorization of power and subsequent vulnerability gives rise to a set of obligations on the part of the fiduciary. The fiduciary must protect and promote the interests of the beneficiary [12]. In short, a fiduciary relationship can be understood as a trust relationship in which one party authorizes another with discretionary powers to act in the other's best interests.

The relationship between a physician-researcher and a patient-subject demonstrates the characteristics of a fiduciary relationship [18]. Patient-subjects lack medical knowledge and prescribing powers; as such, they do not possess the requisite knowledge and ability to ensure that they receive competent medical care [18]. To receive competent medical care, they entrust a fiduciary with more medical knowledge (in this case, the physician-researcher) with discretionary power over their medical interests. This entrustment makes the patient-subject vulnerable and dependent on the judgment and discretion of the physician-researcher. For example, a patient-subject's ability to receive competent medical care while participating in a trial is contingent on a physician-researcher's judgment regarding protocol development, treatment administration, and withdrawal from a study. By consenting to participate in a study, a patient-subject entrusts his or her practical interests to the discretionary power of a physician-researcher and trusts that the physician-researcher will protect and promote these interests to the greatest extent possible within the confines of the research design [18]. As a result of this entrustment, a physician-researcher incurs obligations to the patient-subject, including a duty of care.

The origins of clinical equipoise as an obligation emerging from the relationship between a physician-researcher and a patient-subject interfere with its application to the resolution of tensions associated with harm-benefit assessments in CRTs. A physician-researcher's obligation to ensure that clinical equipoise obtains is derived from the duty of care to patient-subjects. As such, clinical equipoise presupposes the existence of a relationship between a physician-researcher and a patient-subject. Unlike RCTs, CRTs may not involve relationships between physician-researchers and patient-subjects. In CRTs, the units of randomization are often clinics, schools, hospitals, communities, or physician practices rather than individuals suffering from particular conditions or disorders.

Furthermore, CRT interventions are often targeted at health care providers rather than patients and may have only an indirect impact on patient care. In such instances, the research subjects are the health care providers [28]. It is not clear whether the patients of health care providers participating in a CRT need necessarily be considered research subjects. In the above mentioned cases, there is no obvious fiduciary relationship between the physician-researcher and the patient-subject. Consequently, clinical equipoise, which is rooted in the obligations of physician-researchers to individual patient-subjects, is not obviously applicable to CRTs.

## Solutions in the CRT literature

If clinical equipoise is not applicable to CRTs, then what criteria can be used to ensure that groups and individuals are exposed to benefits and harms that stand in reasonable relation while participating in CRTs? Several solutions have been proposed to resolve the ethical tensions arising in CRTs; however, these proposals seem lacking.

To recall, the central ethical problems associated with harm-benefit assessments raised in the CRT literature include the morally appropriate treatment of subjects in the control group of CRTs, and the data accumulation problem. Commentators have proposed three broad solutions to these problems. With respect to concerns about the treatment of control group subjects, commentators have argued that the ethical treatment of control group subjects requires that they be provided with a minimal amount of the experimental intervention and that delayed implementation of the experimental intervention can resolve ethical concerns about undue deprivation of experimental interventions. With respect to the data accumulation problem, commentators have argued that early stopping rules in CRTs can help to resolve ethical concerns about data favoring the experimental treatment generated during a trial. We proceed by critically analyzing these solutions before providing our own.

The first solution proposes to redress injustices created by withholding the experimental intervention from control group subjects by providing control groups with a "minimal level of intervention" [6,8]. According to Glanz and colleagues, "the use of a minimal intervention such as an educational brochure may provide an acceptable level of benefit"[6].

It is not clear that providing a minimal level of benefit to control group subjects can help to ensure the ethical requirement for a reasonable balance of harms and benefits. The authors provide an example of an educational brochure; however, they neglect to provide a definition for a "minimal level of benefit". As such, it is difficult to infer what types of interventions would be sufficient to ensure an ethically appropriate balance of harms and benefits in other CRTs. Furthermore, no argument is provided for why a minimal level of benefit is the appropriate amount. Without a principled argument, it is difficult to understand why this solution results in an ethically appropriate balance between harms and benefits.

The second solution proposes that innovative trial designs in which all subjects eventually receive the experimental intervention can provide subjects in CRTs with a reasonable balance of harms and benefits. Commentators claim that harm-benefit ratios are reasonable if the control group receives the experimental intervention during a later phase or at the conclusion of a trial [6,8]. Similarly, commentators have suggested that ethical concerns about the treatment of subjects in the control group can be resolved by using a stepped wedge design, in which an intervention is provided sequentially to individual subjects or clusters over a number of time periods [29].

These proposals share the common premise that the ethical requirement to balance harms and benefits of participation is met as long as research subjects are provided with the experimental agent at some point during the trial or after its completion. However, a delayed implementation design strategy raises both practical and conceptual difficulties. Practically, it may not be possible to implement the experimental intervention at a later point in a trial. If the cluster population is dynamic, as in an intensive care unit, some patient-subjects may never have the opportunity to receive the experimental intervention.

In addition, delayed implementation of an intervention does not resolve conceptual difficulties. If it is morally impermissible to deprive subjects of an experimental intervention, then it is not clear why delaying implementation of that intervention becomes morally permissible. We are unaware of any ethical argument or principle justifying the moral permissibility of temporarily depriving patient-subjects of treatments until a subsequent point during or after the trial and no such argument is proffered by the above authors. As a result, the success of solutions involving a delayed implementation design relies on the development of a principled argument explaining why the provision of delayed treatment offers subjects a reasonable balance of harms and benefits.

The third solution proposed in the CRT literature attempts to resolve the data accumulation problem by implementing early stopping rules in CRTs. While RCTs are underway, it is common for a data monitoring committee (an independent committee of experts) to safeguard the interests of participants [30]. Traditionally, standard interim tools were not used in CRTs. Commentators argued that the unique structure of CRTs inhibits the use of interim tools, that interventions studied in CRTs are benign, and interim tools are consequently unnecessary [30]. However, trial monitoring and interim reporting have been demonstrated to be necessary and broadly applicable to CRTs [30].

Glanz and colleagues [6] argue that data monitoring committees should evaluate the interim results of CRTs and halt the trials when appropriate. More specifically, interim results can indicate that a trial should be halted early because of adverse effects or because of benefits. According to Glanz and colleagues, when interim results uncover statistically significant and clinically important adverse effects disturbing the balance between the risks and potential benefits of a trial, the trial should be halted [6]. Conversely, when interim findings indicate some success associated with the experimental intervention, a researcher or data monitoring committee is morally required to halt the trial early to provide all subjects with the experimental intervention [6].

Glanz and colleagues' claim that statistically significant and clinically important adverse effects indicate that a CRT should be stopped early is convincing. We agree that the potential scientific gains of a study cannot justify exposing research subjects to an unfair balance of harms and benefits. Furthermore, this belief is reflected in widely accepted guidelines governing the ethics of research with humans. For example, the Declaration of Helsinki indicates that "physicians must immediately stop a study when the risks are found to outweigh the potential benefits" [4].

However, it is less clear that interim findings indicating the superiority of one treatment over another require that a trial be halted to provide all subjects with the more successful intervention. Early stopping based on interim findings may compromise the scientific value of a trial. A trial's ability to generate results important enough to contribute to a change in practice most often requires that a trial run until completion. As a result, it is not clear that early stopping due to a perceived benefit will promote the interests of trial subjects or broader public interests. In short, determining when interim findings indicate that a CRT should not run to completion requires analysis. A successful solution must protect research subjects from undue harm in such a way that permits trials to continue until the point of clinical importance.

In summary, harm-benefit assessments in CRTs raise difficult ethical questions that have not been adequately resolved in the literature on the ethics of CRTs. A principled resolution for the ethical problems in CRTs depends on the applicability of a concept like clinical equipoise. The following section will explore a more successful resolution to the ethical problems raised by harm-benefit assessments in CRTs.

## A different solution: the trust relationship between the state and research subjects

A more successful solution to the analysis of harms and benefits in CRTs involves a principled moral requirement, such as clinical equipoise. In the following section, we argue that the recognition of a new trust relationship can help to situate clinical equipoise as a moral requirement applicable to CRTs as well as RCTs. Miller and Weijer argue that in addition to the trust relationship between physician-researchers and patients-subjects, there exists a trust relationship between the state and research subjects [17]. This trust relationship adds to but does not replace the fiduciary relationship between physician-researcher and patient-subject. Furthermore, it is applicable not only to CRTs but to all clinical trials.

We proceed by elucidating the trust relationship between the state and research subjects and examining its implications for harm-benefit assessments in CRTs and RCTs. The trust relationship between state and research subjects has two central advantages. First, recognition of this trust relationship provides a foundation for clinical equipoise which does not depend on a pre-existing relationship between a physician-researcher and a patient-subject. As a result, this trust relationship permits clinical equipoise to be applied to the resolution of ethical tension in CRTs. Second, it helps to specify the role and obligations of research ethics committees in the ethical analysis of risk in CRT as well as in all RCTs.

The trust relationship between the state and research subjects stems from the social value of clinical research. Clinical research contributes to important public goods, including gains in scientific knowledge and improvements in the quality of health care. This progress depends on the continuation of clinical research, which, in turn, relies on the willingness of citizens to volunteer for research participation. Citizens who volunteer for research participation trust that in return for their contribution to the public good, the state will protect and promote their interests [17]. The trust placed in the state by citizens in combination with the public value derived from research participation incurs a moral obligation on the part of the state to protect the interests of patient-subjects [17]. To put it another way, in the same way that the trust based relationship between physician-researchers and patient-subjects gives rise to obligations for a physician-researcher to act in the interests of a patient-subject, the trust relationship between the state and citizens gives rise to an obligation that the state undertake to protect the interests of individuals participating in research.

To fulfill its obligation to subjects, the state promulgates regulations outlining protections for research subjects and ensures that these regulations are adequately enforced [17]. Specifically, standards for the scientific and ethical permissibility of clinical research are outlined in national guidelines and regulations. These documents delineate the obligations of various parties in clinical research, including both institutions and individual physician-researchers. In addition to these oversight documents, the state enacts regulatory oversight structures, including research ethics committees that are charged with the responsibility of reviewing research proposals to ensure compliance with national standards. Research ethics committees can be understood as fulfilling the state's trust based obligation to protect the liberty and welfare interests of citizens who participate in clinical research [17]. Research ethics committees fulfill this function, in part, by trying to establish a reasonable balance between the harms and benefits of a trial.

Why recognize an additional trust relationship between the state and research subjects? Decisions about whether or not a trial should proceed are determined during ethics review, which is prior to the existence of any relationship between a physician-researcher and patient-subject [17]. At this stage, potential patient-subjects have not been approached regarding study participation; there exist no current relationships but only potential future relationships between physician-researchers and patient-subjects. Consequently, the interests of particular patient-subjects are not yet in view. Although the interests of particular individuals are not yet in view and there are no already existing relationships between physician-researchers and patient-subjects, the ethical conduct of clinical research requires that there be safeguards for the interests of prospective patient-subjects.

Recognizing the trust relationship between the state and research subjects provides a mechanism for justifying protections for research subjects prospectively. In this framework, research ethics committees are responsible for protecting the interests of future patient-subjects [17]. This prospective review includes, but is not limited to, scrutinizing a study for scientific validity, evaluating the recruitment and enrollment criteria, and verifying that adequate procedures are in place to secure informed consent [17].

In summary, ethics review occurs prior to the existence of any particular relationship between a physician-researcher and patient-subject and also to CRTs, in which there may be no physician-researcher and patient-subject relationship. Consequently, research ethics committees play a central role in fulfilling the state's obligation to protect the interests of patient-subjects. In short, the trust relationship between the state and research subjects helps to explain the moral obligations of the state as well as researchers to subjects in RCTs and in CRTs.

## Advantages of the trust relationship between state and research subjects

The trust relationship between the state and research subjects has two central advantages. First, it provides a framework that defines the obligations owed to prospective research subjects at the stage of ethics review. As such, it explains the central role of research ethics committees in protecting subjects. Second, and of central importance to our argument, recognizing the trust relationship between the state and research subjects situates clinical equipoise within a framework applicable to CRTs.

To recall, the problem in applying clinical equipoise to CRTs stems from the understanding of clinical equipoise as an obligation of a physician-researcher to a patient-subject by virtue of the fiduciary relationship. CRTs often involve no relationship between physician-researchers and patient-subjects and, consequently, clinical equipoise does not obviously fit into this framework. Recognizing an independent trust-relationship between the state and research subjects situates clinical equipoise within a framework that does not rely on the relationship between a physician-researcher and patient-subject. As such, it may be relevant to the ethics of harm-benefit assessments in CRTs.

How does clinical equipoise fit into the trust relationship between the state and research subjects? The trust relationship between the state and a group or community of research subjects gives rise to obligations on the part of the state to protect the welfare interests of research subjects. More specifically, patient-subjects have interests in receiving competent medical care. This interest gives rise to a parallel obligation on the part of the state to ensure this protection. To put it another way, as a result of the trust relationship, the state incurs a duty of care towards research subjects. Clinical equipoise emerges as the specification of the duty of care between the state and research subjects. The ethical requirement of clinical equipoise helps to ensure that all research subjects in a trial will receive competent medical care.

To summarize, when understood as the specification of the state's duty of care towards research subjects, clinical equipoise is applicable as a moral requirement for CRTs. Prior to and during these trials, research ethics committees and data monitoring committees help to ensure that clinical equipoise obtains. The implications of the trust relationship between the state and research subjects are important. It helps to resolve ethical questions associated with prospective ethics review in RCTs as well as CRTs, and situates clinical equipoise within a framework applicable to harm-benefit assessments in CRTs. In this framework, research ethics committees and data monitoring committees, in addition to individual physician-researchers, can partially fulfill their ethical obligations towards research subjects by ensuring that clinical equipoise obtains.

### Implications for harm-benefit assessments in CRTs

Interpreting clinical equipoise as a moral requirement relevant to CRTs provides research ethics committees with a principled way of establishing when it is permissible to expose subjects in CRTs to an experimental intervention. As such, it can help provide successful resolutions to the ethical problems arising in the CRT literature concerning the treatment of subjects in control groups and the accumulating data during a CRT.

First, clinical equipoise can help to resolve concerns about unduly depriving control group subjects in CRTs of potentially effective experimental interventions. Glanz and colleagues raise concerns about control group subjects undergoing risks of harm or inconvenience without receiving the benefits of the experimental intervention [6]. Clinical equipoise helps to resolve this tension by providing a clear criterion for the ethical treatment of all research subjects. According to clinical equipoise, exposing subjects to experimental interventions is morally permissible if the evidence supporting the proposed interventions is sufficient that, were it widely known, expert clinicians would disagree about the preferred treatment [3]. The disagreement amongst the relevant community of experts helps to ensure that the effects of the experimental intervention, whether positive or negative, are not known prior to the trial. Without convincing evidence for the beneficial effects of an intervention (which is usually generated at the end of a trial) control group subjects are not being deprived of an intervention known to be effective. In other words, the control group does not receive substandard treatment as long as there is disagreement among the community of expert practitioners about whether the experimental or control intervention is preferable.

Second, Glanz and colleagues argue that when interim analysis shows an improvement in psychosocial or medical outcomes associated with an intervention, "it would then be reasonable to offer the more effective strategy to all communities or participants" [6]. However, this suggestion interferes with a trial's ability to generate useful results. Clinical equipoise resolves this tension without sacrificing the treatment of research subjects or the scientific utility of a trial. While data accumulating prior to and during the course of a trial may seem to favor one treatment over another, clinical equipoise persists until the results are powerful enough to influence the judgment of the community of experts. This type of evidence is most often generated at the end of a trial. As a result, in spite of accumulating evidence suggesting the superiority of the experimental intervention, researchers are ethically justified in continuing a trial to completion.

### Example CRT

In addition to resolving some of the ethical tensions raised in the literature on CRTs, clinical equipoise provides research ethics committees with formal and procedural guidelines for harm-benefit assessments. Through an analysis of the following example, we will demonstrate how clinical equipoise can help to resolve ethical tensions and ambiguity in the assessment of harms and benefits in CRTs.

In one example of a CRT, villages in rural Uganda were randomized either to have or not to have access to group interpersonal psychotherapy (IPT) for depression [31]. Within the villages selected for participation, eligible study participants either self-identified or were identified by others as having symptoms of depression and were assessed according to the diagnostic criteria described in the Diagnostic and Statistical Manual of Mental Disorders, Fourth Edition (DSM-IV). Subjects in villages assigned to the intervention arm received weekly ninety-minute sessions of interpersonal psychotherapy for sixteen weeks [31]. Subjects in villages assigned to the control group received no intervention beyond what was already available to them in their villages. The goals of the study were to test the efficacy of group IPT in reducing symptoms of depression in Uganda and assess the feasibility of depression studies in Uganda [31].

For a CRT to offer a reasonable balance of harms and benefits, it must meet the requirements of clinical equipoise. To determine whether this CRT meets the requirements of clinical equipoise, the trial investigators and research ethics committee, should ask the following questions. Is the evidence supporting the experimental treatment sufficient that, were it widely known, expert practitioners would disagree about the preferred treatment? Are control communities unduly deprived of the experimental treatment?

With respect to this trial, the investigators and research ethics committee should first ask if the evidence supporting the efficacy of group IPT in treating depression in Uganda is sufficient that, were it widely known, expert practitioners would disagree over the efficacy of this intervention? In order for this trial to be ethically permissible, a research ethics committee must find it reasonable to answer yes to this question. The investigators point out that substantial evidence exists, demonstrating the success of psychotherapy in reducing symptoms of depression in developed countries [31], which provides reason to believe that, if implemented successfully, group IPT could prove beneficial to the population of the host country.

However, the local conditions in sub-Saharan Africa and those in which group IPT was developed differ dramatically and these variations may complicate the successful implementation of the therapeutic strategies required for group IPT [31]. For example, local communities are often hesitant to communicate directly about sensitive issues. This poses a difficult obstacle for psychotherapy, which relies on discussion therapy [31]. In short, evidence exists suggesting the potential success of group IPT in reducing symptoms of depression; however, the efficacy and the feasibility of implementing group IPT in sub-Saharan Africa are questionable. To put it another way, experts would likely disagree over the success or feasibility of implementing IPT in sub-Saharan Africa, thereby meeting one moral requirement of clinical equipoise.

The second question investigators and research ethics committees should ask is whether the control communities are being unduly deprived of group IPT in this trial? Undue deprivation of an experimental intervention occurs if a patient-subject's participation in a trial restricts access to therapies which would otherwise be available as part of standard care. At the outset of the trial, the standard of care for treating depression in sub-Saharan Africa did not include psychotherapy. Traditional healers were available to village inhabitants who sought their expertise; however, there was no intervention typically administered for the treatment of depression. Control group subjects were not discouraged from visiting traditional healers or taking any measures available to them to relieve their depression [31]. Instead, the trial sought to measure the success of group IPT in comparison with the usual treatment [31]. Given that subjects were not denied standard treatment available in Uganda and that the efficacy of group IPT remains uncertain in these populations, control subjects were not unduly deprived of the experimental intervention. In other words, it seems reasonable for a research ethics committee to find that this CRT meets the moral requirement of clinical equipoise.

## Conclusion

Harm-benefit assessments raise difficult problems for CRTs. These problems resemble the ethical problems in RCTs that are resolved by clinical equipoise; however, the unique structure of CRTs resists the application of clinical equipoise as the moral principle for harm-benefit assessments. Problems in applying clinical equipoise to CRTs arise from the origins of clinical equipoise as a duty of care emerging from the fiduciary relationship between physician-researchers and patient-subjects. We argue that situating clinical equipoise within a trust relationship between the state and a community of subjects provides a framework in which clinical equipoise can be applied to CRTs. Finally, applying clinical equipoise to the ethical analysis of risk in CRTs can help to resolve the tensions arising in harm-benefit assessments and provide research ethics committees with criteria for the ethical treatment of subjects in control and experimental arms of a trial.

## Note

We have created a Wiki webpage to facilitate an open discussion about the ideas expressed in this and other papers published in the series on ethical issues in CRTs. Please enter your thoughts and comments at http://crtethics.wikispaces.com.

## Competing interests

AB, JCB, ADM, RS, MT, and CW: None declared

RB, AD, MPE, JMG, and MZ have all submitted cluster trial protocols to ethics committees and had difficulty explaining to them the differences between cluster randomized trials and individual patient clinical trials.

## Authors' contributions

AB, CW, ADM, and JMG contributed to the conception and design of the manuscript. AB and CW led the writing of the manuscript. All authors commented on sequential drafts and approved the final version.

## References

[B1] Weijer C Grimshaw JM Taljaard M Binik A Boruch R Brehaut JC Donner A Eccles MP Gallo A McRae AD Saginur R Zwarenstein M Ethical issues posed by cluster randomized trials in health research Trials 2011 12 100 10.1186/1745-6215-12-100 21507237 PMC3107798

[B2] Weijer C Miller PB When are research risks reasonable in relation to anticipated benefits? Nat Med 2004 10 6 570 3 10.1038/nm0604-570 15170195

[B3] Freedman B Equipoise and the ethics of clinical research N Engl J Med 1987 317 3 141 5 10.1056/NEJM198707163170304 3600702

[B4] World Medical Association Declaration of Helsinki: Ethical Principles for Medical Research involving Human Subjects 2008 http://www.wma.net/en/30publications/10policies/b3 [date accessed: April 17, 2011]

[B5] Taljaard M Weijer C Grimshaw JM Belle Brown J Binik A Boruch R Brehaut JC Chaudhry SH Eccles MP McRae A Zwarenstein M Donner A Ethical and policy issues in cluster randomized trials: rationale and design of a mixed methods research study Trials 2009 10 61 10.1186/1745-6215-10-61 19638233 PMC2725043

[B6] Glanz K Rimer BK Lerman C Coughlin S, Beauchamp T Ethical issues in the design and conduct of community-based intervention studies Ethics and Epidemiology 1996 Oxford: Oxford University Press

[B7] Hutton JL Are distinctive ethical principles required for cluster randomized controlled trials? Stat Med 2001 20 3 473 88 10.1002/1097-0258(20010215)20:3<473::AID-SIM805>3.0.CO;2-D 11180314

[B8] Klar N Donner A D'Agostino RB, Sullivan L, Massaro J Ethical challenges posed by cluster randomization Wiley Encyclopedia of Clinical Trials 2008 Hoboken, NJ: Wiley Interscience

[B9] Medical Research Council Cluster Randomised Trials: Methodological and Ethical Considerations 2002 http://www.mrc.ac.uk/Utilities/Documentrecord/index.htm?d=MRC002406 [date accessed: April 17, 2011]

[B10] Ware J Investigating therapies of potentially great benefit: ECMO Statistical Science 1989 4 4 298 306 10.1214/ss/1177012384

[B11] Shaw LW Chalmers TC Ethics in cooperative clinical trials Ann N Y Acad Sci 1970 169 2 487 95 10.1111/j.1749-6632.1970.tb54759.x 4907495

[B12] Miller PB Weijer C Rehabilitating equipoise Kennedy Inst Ethics J 2003 13 2 93 118 10.1353/ken.2003.0014 14569997

[B13] U.S. National Bioethics Advisory Commission Ethical and Policy Issues in Research Involving Human Participants. Bethesda, Maryland 2000

[B14] Angell M The ethics of clinical research in the Third World N Engl J Med 1997 337 12 847 9 10.1056/NEJM199709183371209 9295243

[B15] Crouch RA Arras JD AZT trials and tribulations Hastings Cent Rep 1998 28 6 26 34 10.2307/3528266 9868608

[B16] Miller FG Brody H A critique of clinical equipoise. Therapeutic misconception in the ethics of clinical trials Hastings Cent Rep 2003 33 3 19 28 10.2307/3528434 12854452

[B17] Miller PB Weijer C Trust based obligations of the state and physician-researchers to patient-subjects J Med Ethics 2006 32 9 542 7 10.1136/jme.2005.014670 16943338 PMC2563395

[B18] Miller PB Weijer C Fiduciary obligation in clinical research J Law Med Ethics 2006 34 2 424 40 10.1111/j.1748-720X.2006.00049.x 16789965

[B19] Djulbegovic B Articulating and responding to uncertainties in clinical research J Med Philos 2007 32 2 79 98 10.1080/03605310701255719 17454416

[B20] Veatch RM Indifference of subjects: an alternative to equipoise in randomized clinical trials Soc Philos Policy 2002 19 2 295 323 10.1017/S0265052502192120 12678091

[B21] Ashcroft R Equipoise, knowledge and ethics in clinical research and practice Bioethics 1999 13 3-4 314 26 10.1111/1467-8519.00160 11657242

[B22] Karlawish JH Lantos J Community equipoise and the architecture of clinical research Camb Q Healthc Ethics 1997 6 4 385 96 10.1017/S0963180100008136 9292216

[B23] Gifford F Community-equipoise and the ethics of randomized clinical trials Bioethics 1995 9 2 127 48 10.1111/j.1467-8519.1995.tb00306.x 11653056

[B24] Weijer C Shapiro S Glass KC Enkin MW Clinical equipoise and not the uncertainty principle is the moral underpinning of the randomised controlled trial BMJ 2000 321 7263 756 9 10.1136/bmj.321.7263.756 10999914 PMC1127868

[B25] Friedman L Furberg C DeMets D Fundamentals of Clinical Trials 1998 New York: Springer 246 83

[B26] Katz J Experimentation with Human Beings 1972 New York: Russell Sage Foundation

[B27] Levine R Ethics and Regulations of Clinical Research 1988 New Haven: Yale University Press

[B28] Mann H Reyes M Identifying the human research subject in cluster randomized controlled trials IRB 2008 30 5 14 8 18953772

[B29] Brown C Hofer T Johal A Thomson R Nicholl J Franklin BD Lilford RJ An epistemology of patient safety research: a framework for study design and interpretation. Part 4. One size does not fit all Qual Saf Health Care 2008 17 3 178 81 10.1136/qshc.2007.023663 18519623

[B30] Donner A Klar N Design and Analysis of Cluster Randomization Trials in Health Research 2000 London: Arnold

[B31] Bolton P Bass J Neugebauer R Verdeli H Clougherty KF Wickramaratne P Speelman L Ndogoni L Weissman M Group interpersonal psychotherapy for depression in rural Uganda: a randomized controlled trial JAMA 2003 289 23 3117 24 10.1001/jama.289.23.3117 12813117

